# 

**Published:** 1898-06-25

**Authors:** 


					THE HOSPITAL. "The standard of highest purity.'1-?Lancet June 25th, 1898.
CADBURY'S TOT CADBURY'S
COCOA inll M COCOA
ABSOLUTELY PURE. r _ l NO DRUGS OR ALKALIES USED.
a
^^^MOLASCOW WrsTERH INFIRMARY
No. 613. Vol. XXIV. [JSSUG Saturday,
June 25th, 1898.
[-Sub?criptionTemH,] pRICE TWOPENCE.

				

